# Genome-Wide DNA Methylation and Gene Expression Profiling Characterizes Molecular Subtypes of Esophagus Squamous Cell Carcinoma for Predicting Patient Survival and Immunotherapy Efficacy

**DOI:** 10.3390/cancers14204970

**Published:** 2022-10-11

**Authors:** Yulong Zheng, Qiqi Gao, Xingyun Su, Cheng Xiao, Bo Yu, Shenglin Huang, Yifeng Sun, Sheng Wu, Yixin Wo, Qinghua Xu, Nong Xu, Hui Yu

**Affiliations:** 1 Department of Medical Oncology, The First Affiliated Hospital, School of Medicine, Zhejiang University, Hangzhou 310003, China; 2 Department of Pathology, The First Affiliated Hospital, School of Medicine, Zhejiang University, Hangzhou 310003, China; 3 Department of Thoracic Medical Oncology, Fudan University Shanghai Cancer Center, Shanghai 200032, China; 4 Department of Oncology, Shanghai Medical College, Fudan University, Shanghai 200032, China; 5 The Shanghai Key Laboratory of Medical Epigenetics, Fudan University Shanghai Cancer Center, Institutes of Biomedical Sciences, Fudan University, Shanghai 200032, China; 6 The Canhelp Genomics Research Center, Canhelp Genomics Co., Ltd., Hangzhou 311199, China; 7 The Institute of Machine Learning and Systems Biology, College of Electronics and Information Engineering, Tongji University, Shanghai 201804, China; 8 Xuzhou Engineering Research Center of Medical Genetics and Transformation, Department of Genetics, Xuzhou Medical University, Xuzhou 221004, China

**Keywords:** esophagus squamous cell carcinoma, molecular subtype, prognosis, immune checkpoint inhibitors, gene expression signature

## Abstract

**Simple Summary:**

Esophageal squamous cell carcinoma (ESCC) represents roughly 85–90% of all esophageal carcinoma patients in China. Immunotherapy is used to treat an increasing number of ESCC patients in clinical practice. This study aims to understand the molecular heterogeneity and the tumor immune microenvironment of ESCC for designing novel immunotherapies to improve response and outcomes. We identified two molecular subtypes associated with prognosis, immune-related pathways, and tumor microenvironment. In an independent cohort of Chinese ESCC patients treated with immunotherapy, the response rate of the S1 subtype is significantly higher than the S2 subtype. These findings provide a new perspective on the molecular subtyping for ESCC and a biological rationale for novel therapeutic intervention in a specific subgroup of ESCC that could potentially be translated into clinical practice both diagnostically and therapeutically to benefit ESCC patients.

**Abstract:**

Background: Immunotherapy is recently being used to treat esophageal squamous cell carcinoma (ESCC); however, response and survival benefits are limited to a subset of patients. A better understanding of the molecular heterogeneity and tumor immune microenvironment in ESCC is needed for improving disease management. Methods: Based on the DNA methylation and gene expression profiles of ESCC patients, we identify molecular subtypes of patients and construct a predictive model for subtype classification. The clinical value of molecular subtypes for the prediction of immunotherapy efficacy is assessed in an independent validation cohort of Chinese ESCC patients who receive immunotherapy. Results: We identify two molecular subtypes of ESCC (S1 and S2) that are associated with distinct immune-related pathways, tumor microenvironment and clinical outcomes. Accordingly, S2 subtype patients had a poorer prognosis. A 15-gene expression signature is developed to classify molecular subtypes with an overall accuracy of 94.7% (89/94, 95% CI: 0.880–0.983). The response rate of immunotherapy is significantly higher in the S1 subtype than in the S2 subtype patients (68.75% vs. 25%, *p* = 0.028). Finally, potential target drugs, including mitoxantrone, are identified for treating patients of the S2 subtype. Conclusions: Our findings demonstrated that the identified molecular subtypes constitute a promising prognostic and predictive biomarker to guide the clinical care of ESCC patients.

## 1. Introduction

Esophageal carcinoma was diagnosed in 604,100 patients and was associated with approximately 544,076 deaths worldwide in 2020, ranking seventh in incidence and sixth in mortality [1]. Esophageal carcinoma has two most common histological subtypes, including esophageal adenocarcinoma (EAC) and esophageal squamous cell carcinoma (ESCC). In China, ESCC represents roughly 85–90% of all esophageal carcinoma patients, with heavy drinking, smoking, high body mass index and a low-fruit diet among the key risk factors [2,3].

For early-stage ESCC, surgery is the most effective treatment option, but still, 40% of patients eventually have a relapse [4,5]. ESCC often presents imperceptible clinical symptoms at an early stage, the majority of patients diagnosed with advanced-stage ESCC [2]. Currently, standard first-line treatment options for advanced ESCC are limited, mostly based on fluoropyrimidine and platinum themes [6]. Clinical benefits of fluoropyrimidine/platinum-based chemotherapy remain limited with a median overall survival (OS) of less than one year [7,8]. Recently, immune checkpoint inhibitors (ICIs), particularly programmed death-1/programmed death-ligand 1 (PD-1/PD-L1) inhibitors, have shown antitumoral activity in patients with advanced ESCC. Multiple randomized studies, including CheckMate 648, JUPITER-06, ESCORT-1st and KEYNOTE-590, have demonstrated that the clinical benefit of ICIs in combination with chemotherapy is superior to chemotherapy alone in ESCC patients [9,10,11,12].

PD-L1 is commonly elevated in tumor cells and is now currently considered a predictive marker for immunotherapy in solid tumors [13]. The PD-L1 expression in tumor cells is enriched in ESCC patients, with an expression of 1% or greater observed in approximately 30% to 49% of patients [12,14]. Nonetheless, the predictive value of PD-L1 status in ESCC is still controversial [15]. In the CheckMate 648 study, the PD-L1 status was associated with the efficacy of ICIs in ESCC patients. For patients with tumor-cell PD-L1 of 1% or higher, nivolumab plus chemotherapy was found to have a significant progression-free survival benefit when compared to chemotherapy alone [12]. However, in Chinese ESCC patients, PD-L1 was not a predictive biomarker for clinical benefit. The ESCORT-1st trial showed no statistically significant and definite correlation between PD-L1 status and the efficacy of camrelizumab plus chemotherapy in Chinese ESCC patients [10]. In another Chinese ESCC cohort, the JUPITER-06 study found that toripalimab-combined chemotherapy is efficacious irrespective of PD-L1 status [11].

Thus, a better understanding of the molecular heterogeneity and tumor microenvironment in ESCC is essential for designing novel immunotherapies to improve response and prognosis. Here, we performed multi-omics analyses to integrate gene expression profiles and DNA methylation profiles of ESCC samples and revealed two distinct molecular subtypes (subtypes S1 and S2). We found that each molecular subtype was associated with different gene expression profiling, the composition of tumor-infiltrating immune cells, as well as clinical outcomes. A machine learning-based gene expression signature was developed to enable to assign molecular subtype status for individual patients. We further validated the predictive value of the defined molecular subtypes in an independent cohort of Chinese ESCC patients, all receiving immunotherapy plus chemotherapy. Beyond the subtype diagnostic and prognostic model, potential agents specific for subtype S2 were predicted, which could open up new therapeutic options for improving the treatment efficacy of ESCC patients.

## 2. Materials and Methods

### 2.1. Acquisition of TCGA Cohort and Multiomics Data Processing

The legacy archive information of clinical and transcriptomic data of 94 ESCC patients from The Cancer Genome Atlas project (TCGA cohort) were retrieved using R package TCGAbiolinks (version 2.16.4) from the Genomic Data Commons (GDC) Data platform. The obtained transcriptomic data in RSEM format were normalized by Z-score and Log2 transformation. DNA methylation data profiled with Illumina Human Methylation 450 microarray were downloaded from the UCSC Xena browser (https://xenabrowser.net/, accessed on 21 January 2022). The DNA methylation levels (as beta values) were normalized by Z-score transformation. The gene expression data and DNA methylation data were normalized using the data.normalization function of the R package CancerSubtypes (version 1.14.0). Noninformative genes and CpG sites with zero variance cross samples were excluded.

### 2.2. Prognostic Features Selection and Survival Analysis

With the TCGA cohort, a univariate Cox proportional hazards regression analysis was performed to screen genes and CpG sites associated with 94 ESCC patients’ OS status. The Cox model estimated the hazard ratio (HR), confidence interval and Cox *p*-value of each gene and CpG site. HR greater than one indicates that the gene expression or methylation of the CpG site is positively correlated with patient risk, whereas HR less than one indicates a negative correlation. The OS of molecular subtypes was evaluated using Kaplan–Meier curves and log-rank tests. To assess the prognostic utility of molecular subtypes, a univariant Cox proportional regression model was employed. The association between molecular subtypes and survival outcomes was calculated by a multivariate Cox proportional regression model. A significance threshold of 0.05 was applied.

### 2.3. Similarity Network Fusion and Consensus Clustering Analysis

Previous study suggested that both methylation and expression profiles contribute significantly and differently to define tumor molecular subtypes [16]. Two data types provide distinct but complementary signals that improve over single-modality analyses. Therefore, an integrated approach that allows a unified view of the underlying groups would be valuable in elucidating heterogeneity within subgroups. Herein, we conducted the Similar Network Fusion and Consensus Clustering (SNF-CC) analysis on 94 ESCC samples using both gene expression and DNA methylation data. We combined the matrix of 1158 genes’ expression data and 24,451 CpGs’ methylation data for integrative analysis. The ExecuteSNF.CC function implanted in R package CancerSubtypes (version 1.14.0) was used to run SNF-CC analysis. The following detailed parameters were designed to ensure a compromise between high stability and low ambiguity: clusterNum = 2~10, K = 20, alpha = 0.5, t = 20, maxK = 10, pItem = 0.8 and reps = 500. The appropriate number of clusters was selected by the ‘estimateNumberOfClustersGivenGraph’ function implanted in R package SNFtool (version 2.3.1) [17]. Moreover, we use a similarity matrix to calculate the silhouette width, which indicates how closely a specimen is matched to its recognized subtype when compared to other subtypes.

### 2.4. Assessment of Tumor-Infiltrating Immune Cells

CIBERSORT is a deconvolution algorithm that can be used for elucidating the tumor-infiltrating immune cells according to gene expression data [18]. The immune cells included T cells (T cells CD8, T cells CD4 naive, T cells CD4 memory resting, T cells CD4 memory activated, T cells follicular helper, T cells regulatory (Tregs) and T cells gamma delta), B cells (B cells naive, B cells memory, plasma cells), NK cells (NK cells resting, NK cells activated), monocytes, macrophages M0, M1 and M2, dendritic cells resting, dendritic cells activated, mast cells resting, mast cells activated, eosinophils and neutrophils [19]. All analyzed immune cell type fractions for each tumor sample added up to one. Recently, CIBERSORT has been extended to the ‘absolute mode’ (referred to as ‘CIBERSORT-ABS’), which provides a score that can be compared between both samples and cell types [19]. In the current study, CIBERSORT-ABS analysis was conducted to estimate the fraction of tumor-infiltrating immune cells for each 94 ESCC samples.

### 2.5. Identification of Differentially Expressed Genes between Molecular Subtypes and Functional Enrichment Analysis

Differentially expressed gene analysis was conducted using the BRB-ArrayTools software (https://brb.nci.nih.gov/BRB-ArrayTools/, accessed on 25 February 2022) [20]. The univariate “Two-sample *t*-test” was performed to assess the statistical significance *p*-value of gene expression difference. Adjusted *p*-values for significant genes were computed based on 10,000 random permutations. Genes with adjusted *p*-values < 0.001 were considered to be significantly differentially expressed between two subtypes. Furthermore, Gene Ontology (GO) and Kyoto Encyclopedia of Genes and Genomes (KEGG) enrichment analysis of differentially expressed genes were analyzed using the LYNX bioinformatics tool (https://lynx.cri.uchicago.edu/, accessed on 25 February 2022) [21]. The statistical significance *p*-value was evaluated by hypergeometric test and adjusted by Benjamini-Hochberg (BH) correction. Biological categories and pathways with BH adjusted *p*-value ≤ 0.05 were represented significantly enriched.

### 2.6. Signature Gene Identification and Classification Model Construction

Feature selection, model training and evaluation were conducted using R package caret (version 6.0-90) [22]. Using the 94 samples of TCGA cohort, a linear support vector machine (SVM) model was applied to distinguish different subtypes based on the 1158-gene expression data. Briefly, recursive feature elimination with a 10-fold cross-validation process was used to screen signature genes after data standardization and normalization [23]. The prediction score was calculated by scaling the distance to the SVM classifier boundary into the range from 0 to 1 using the sigmoid function. To evaluate the diagnostic performance of the identified gene signature, the prediction scores estimated by the SVM classifier and the binary subtype labels were used to calculate the area under the receiver operating characteristic curve (AUC). The cut-off value of the Youden index (prediction score ≥ 0.5) was used to categorize patients as belonging to different subtypes.

### 2.7. Acquisition of Chinese ESCC Patient Samples

To validate the performance of the established model in a clinical setting, we collected 36 Chinese ESCC patients who had received anti-PD-1/PD-L1 therapy in combination with chemotherapy between January 2018 and December 2020 from The First Affiliated Hospital, School of Medicine, Zhejiang University. All patients had histologically confirmed esophageal squamous cell carcinoma and had measurable disease. All tumor samples were classified based on the American Joint Committee on Cancer (AJCC) tumor-node-metastasis (TNM) system (7 edition). According to Response Evaluation Criteria in Solid Tumors (RECIST, version 1.1), imaging studies were used to gauge the tumor response. Patients were classified as either responder if they had a complete response (CR) or partial response (PR) or non-responders if they had stable (SD) or progressive disease (PD) [24]. Detailed clinical information on age, gender, smoke, alcohol, histopathological factors and anti-tumor treatments was obtained from the medical records. This study was approved by the Institutional Review Board of The First Affiliated Hospital, School of Medicine, Zhejiang University (2022-346) in concordance with the Declaration of Helsinki.

### 2.8. Nucleic Acid Extraction and Gene Expression Profiling

Samples were obtained as formalin-fixed paraffin-embedded (FFPE) tissue and stored under ordinary temperatures until processed for the purification of nucleic acids. Total RNAs were isolated according to the instructions using an FFPE Total RNA Extraction Kit (Canhelp Genomics Co., Ltd., Hangzhou, China). Before constructing the RNA-seq libraries, total RNAs were processed with DNase I (NEB) to eliminate DNA. The SMART cDNA synthesis technology (Clontech, San Jose, CA, USA) was used to prepare strand-specific RNA-seq libraries. The cDNA was pre-amplified and the ribosomal and mitochondrial cDNA were depleted by CRISPR/Cas9 system. Purified dsDNA underwent an additional PCR amplification (13 cycles). Qubit (Thermo Fisher Scientific, Pleasanton, CA, USA) and Qsep100 (BiOptic, New Taipei, Taiwan, China) were used to control the quality of the libraries before sequencing on the Illumina sequencing platform (Nova) using a 150 bp paired-end run. Sequencing reads from RNA-seq data were aligned using the spliced read aligner HISAT2, which was supplied with the Ensembl human genome assembly (Genome Reference Consortium GRCh38) as the reference genome. The fragments per kilobase of transcript per million mapped reads (FPKM) were used to calculate gene expression levels.

### 2.9. Prediction of Immune Checkpoint Blockade Therapy Response and Drug Repurposing

To predict patients’ responses to immunotherapies in the validation cohort of 36 ESCC patients, we used two immune-related analysis tools applied to samples from the validation cohort. First, the Tumor Immune Dysfunction and Exclusion algorithm (TIDE) is a computational method for predicting response of ICIs therapy based on gene expression profiling [25]. Patients with high TIDE scores indicate increased potential for tumor immune escape and resistance to immunotherapy. Second, the Immune Cell Abundance Identifier algorithm (ImmuCellAI) is able to estimate the abundance of 24 immune cell types as well as predict patients’ response to ICIs therapy based on gene expression datasets [26]. The 24 immune cell types consist of 18 T-cell subtypes and 6 other immune cell types (B cell, natural killer cell, monocyte cell, macrophage cell, neutrophil cell and dendritic cell). Furthermore, we investigated potential small molecule drugs by searching the L1000 Connectivity Map Resource by “Enrichr” enrichment analysis tool (https://maayanlab.cloud/Enrichr/, accessed on 18 March 2022) [27].

## 3. Results

### 3.1. Integrative Analysis of DNA Methylation and Gene Expression Profiles Reveals Two Molecular Subtypes of ESCC 

**Figure 1:**
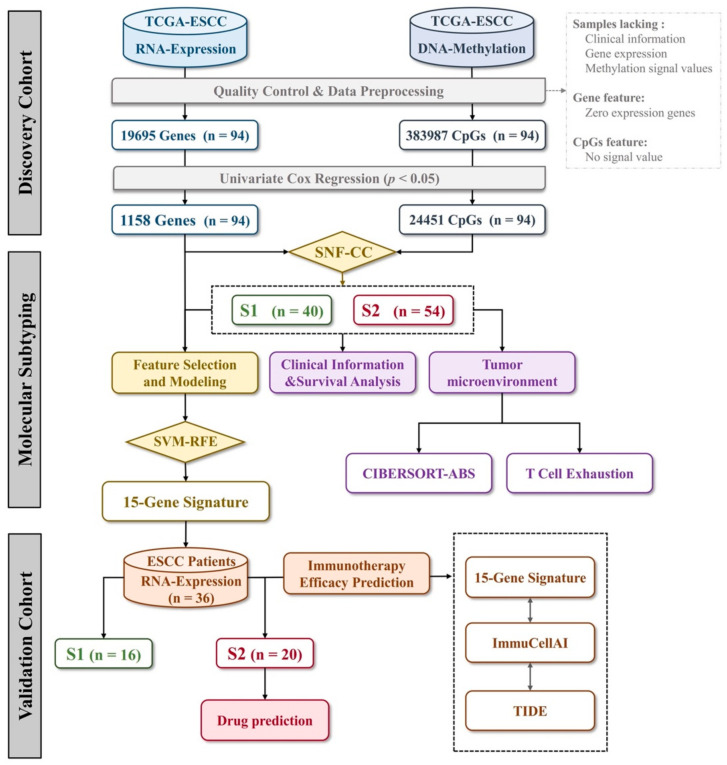
Study design.

**Figure 2:**
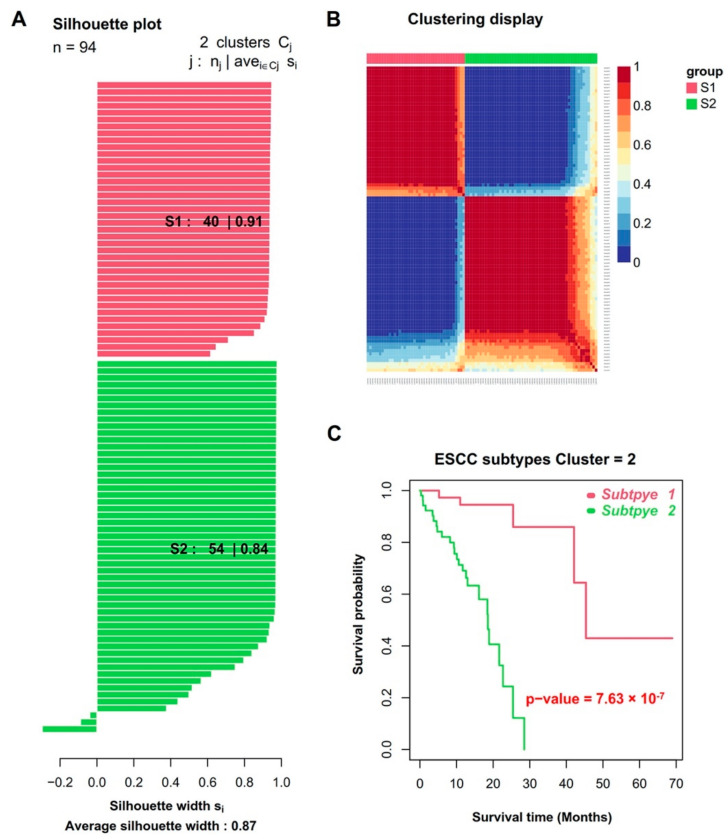
Identification of two subtypes using SNF-CC algorithm. (**A**) Silhouette width plots of the subtypes for k = 2. (**B**) Heatmap representation of the sample-to-sample fused network data sorted by cluster for k = 2. Sample similarity is represented by red (high similar) to blue (less similar) coloring inside the heatmap. (**C**) Kaplan–Meier curves comparing overall survival between S1 and S2 subtypes.

The workflow to identify, test and validate molecular subtypes of ESCC is presented in Figure 1. Paired gene expression and DNA methylation data of 94 ESCC samples were retrieved from the TCGA database. The sample clinical information, including age, gender, race, tumor characteristics, radiation treatment and survival status, is presented in Table 1. For these samples, we first performed the univariate Cox regression analysis on both gene expression and DNA methylation data. The results revealed that a set of 1158 genes and 24,451 CpGs were significantly related to patients’ OS outcomes (Cox *p*-value < 0.05). We then looked into whether integrative data analysis could be used to divide these ESCC samples into clinically relevant molecular subtypes. We applied SNF to integrate the prognostic gene expression and DNA methylation data (1158 genes and 24,451 CpGs related to prognosis), followed by consensus clustering ranging from 2 to 10 groups. The optimal number of clusters was two according to the results of the ‘estimateNumberOfClustersGivenGraph’ function. At k = 2, two distinct subtypes (S1 and S2) were clearly identified with the maximal average silhouette width (Figure 2A). There were 40 and 54 tumor specimens each for the S1 and S2 subtypes, respectively (Figure 2B). The clinicopathological characteristics between the S1 and S2 subtypes showed no significant differences (Table 1). Compared to patients in the S1 subtype, S2 subtype patients had inferior OS outcomes (Figure 2C). The median OS for the S1 and S2 subtypes was 45.4 and 18.6 months, respectively (log-rank *p*-value = 7.63 × 10^−7^). The univariate Cox regression analysis showed that the molecular subtypes were significantly associated with OS (HR = 13.5, Cox *p*-value = 4.91 × 10^−5^, Table 2). The multivariate Cox regression analysis further confirmed that the defined molecular subtypes could be used as a prognostic factor independent of other clinicopathological parameters (HR = 51.6, Cox *p*-value = 2.5 × 10^−3^, Table 2).

### 3.2. Revealing the Relationship between Molecular Subtypes and Tumor Microenvironment

**Figure 3:**
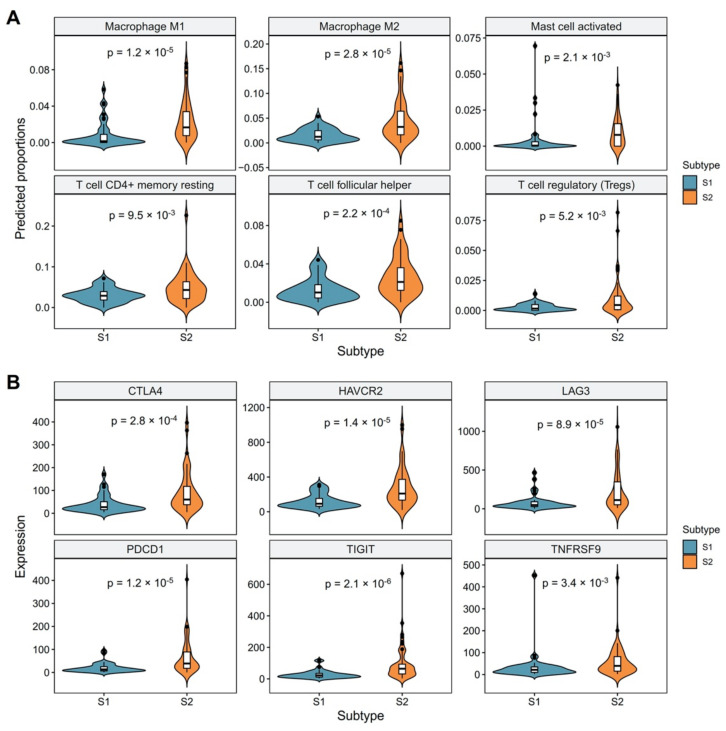
The distinct immune characteristics between two molecular subtypes in the TCGA cohort. (**A**) The predicted proportions of macrophages, activated mast cell, T cell CD4+ memory resting, T cell follicular helper and regulatory T cells (Tregs) between the S1 and S2 subtypes. (**B**) The expression levels of *CTLA4*, *LAG3*, *PDCD1*, *HAVCR2*, *TIGIT* and *TNFRSF9* genes between the S1 and S2 subtypes. Wilcoxon rank sum test served as the statistical significance test.

Owing to the prognostic significance of molecular subtypes, we speculated that two subtypes might be relative to immune activities and tumor microenvironment (TME). We applied the CIBERSORT ABS algorithm to assess the abundance of 22 distinct immune cell types for each sample and between two molecular subgroups. As shown in Figure 3A, the high-risk group (S2 subtype) exhibited increased predicted proportions of regulatory T cells (Tregs), T cell CD4+ memory resting, T cell follicular helper, macrophages and activated mast cells (Wilcoxon *p*-value < 0.01). Previous studies have proved that Tregs might hasten the development of ESCC, and both Tregs and macrophages were relevant to poor prognosis in ESCC patients [28,29,30]. Similarly, it has been reported that high mast cell density is a predictor of poor survival in ESCC patients [31]. Effector T cells in the TME are inclined to high expression levels of numerous inhibitory receptors, including PD-1, Hepatitis A Virus Cellular Receptor 2 (*HAVCR2*, also known as *TIM3*), T cell immunoreceptor with Ig and ITIM domains (*TIGIT*) and lymphocyte activating 3 (*LAG3*), which are considered to be symptoms of a dysfunctional state, well recognized as T cell exhaustion [32]. We, therefore, sought to further investigate the relationship between the molecular subtypes and the mRNA expression levels of multiple inhibitory receptors. Interestingly, we observed several inhibitory receptors, including *CTLA4*, *LAG3*, *PDCD1*, *HAVCR2*, *TIGIT* and *TNFRSF9*, were significantly upregulated in patients of the high-risk group (Two-sample *t*-test *p*-value < 0.05, Figure 3B). These findings suggest that poorer outcomes for the high-risk group (S2 subtype) might be partially caused by the immunosuppressive microenvironment and high T cell exhaustion. Because T cells are the direct target for several immunotherapies, high T cell exhaustion may also influence the S2 subtype patients’ response to antitumor immunotherapies.

### 3.3. Identification and Evaluation of a 15-Gene Signature for Subtype Classification

Using BRB-ArrayTools software, we performed differential gene expression analysis between the S1- and S2-subtype samples. When compared to the S1 subtype, a total of 289 and 16 genes were significantly up- and down-regulated in the S2 subtype, respectively (Supplementary Table S1). By using the LYNX bioinformatics tool, GO category and KEGG pathway enrichment analyses were performed to further investigate the potential biological significances of these differentially expressed genes. Interestingly, GO analysis revealed that the differentially expressed genes were primarily enriched in immune-related processes, such as “regulation of immune system process”, “regulation of lymphocyte activation”, “immune effector process”, “regulation of response to stimulus” and so on (Supplementary Table S2). In addition, we found that the significant KEGG pathways were also enriched in immune-related signal pathways, including “Natural killer cell mediated cytotoxicity”, “Cell adhesion molecules (CAMs)”, “Cytokine-cytokine receptor interaction”, “Antigen processing and presentation” and so on (Supplementary Table S3).

**Figure 4:**
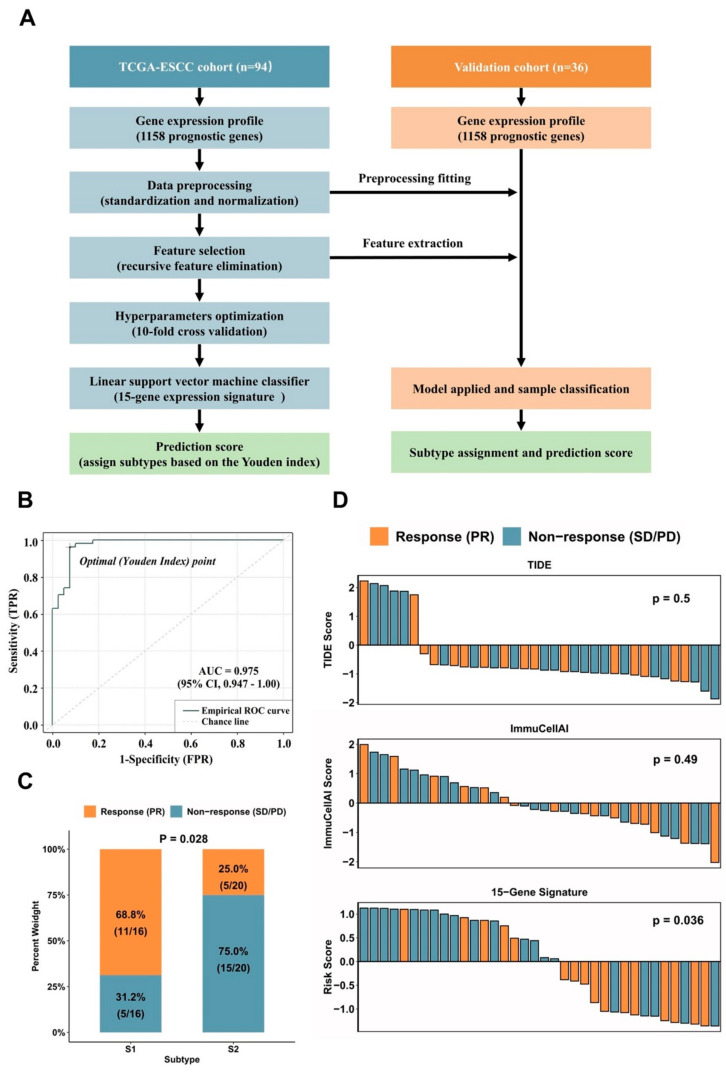
Identification and validation of a 15-gene expression signature. (**A**) Flow chart of the 15-gene expression signature identification. (**B**) The ROC curve of the 15-gene expression signature in the validation set. (**C**) Response rates between S1 and S2 subtypes. (**D**) Waterfall plots of prediction scores of TIDE, ImmuCellAI and 15-gene expression signature across 36 ESCC patients treated with ICIs. ROC, receiver operating characteristic; AUC, area under the curve; ESCC, esophagus squamous cell carcinoma; ICIs, immune checkpoint inhibitors.

**Figure 5:**
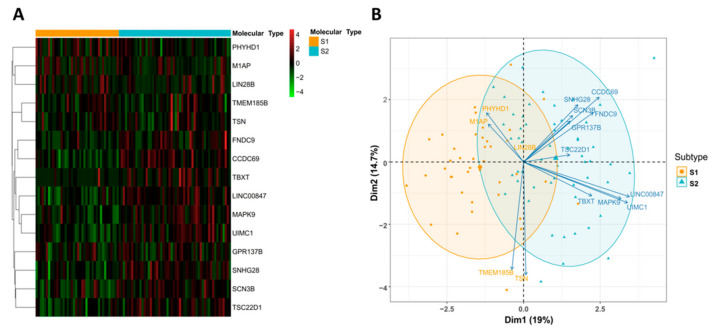
(**A**) Hierarchical clustering analysis of 15 genes in two subtypes. (**B**) Principal component analysis of 15 genes between two subtypes.

We then sought to identify subtype diagnostic signatures, which may enable us to assign the defined molecular subtypes to individual clinical samples. We used the expression levels of 1158 prognostic genes in 94 samples as a training set. The Recursive feature elimination-SVM (RFE-SVM) algorithm was adopted to construct a classification model (Figure 4A). A 10-fold cross-validation process was repeated 100 times to determine the best-performing features and hyperparameters. Intriguingly, a linear SVM model consisting of 15 genes was developed to classify a sample belonging to subtype S1 or S2. Subsequently, a prediction score indicating the probability of the sample belonging to subtypes S1 or S2 was calculated by the linear SVM model. Of the 94 samples, the 15-gene expression signature classified 39 as subtype S1 and 55 as subtype S2. The overall accuracy reached 94.7% (89/94, 95% CI: 0.880–0.983). The ROC curve further confirmed that the identified diagnostic signature had a robust accuracy for classifying subtype S1 and S2 (AUC = 0.975, 95% CI: 0.947–1.00, Figure 4B). Among the selected gene panels, 5 were up-regulated in subtype S1 and 10 were up-regulated in subtype S2 (Supplementary Table S4). The hierarchical clustering and principal component analysis of the expression levels of 15 genes are shown in Figure 5. Similar data analysis was also performed with the DNA methylation data. The classification accuracy of the methylation-based model was a bit lower compared with the accuracy of the gene expression-based model (93.6% vs. 94.7%). When combining the gene expression data and DNA methylation data together, the overall accuracy of the hybrid model was not further improved compared with the gene expression-based model alone.

### 3.4. Independent Validation of the Predictive Power of Molecular Subtypes for Immunotherapy Efficacy

We further validated the predictive power of molecular subtypes in an independent cohort of 36 Chinese ESCC patients who had received PD-1/PD-L1 therapy in combination with chemotherapy. Detailed demographic, clinical and pathological information for patients is summarized in Table 3 The 15-gene expression signature was applied to the 36 samples and assigned 16 samples as S1 subtype (44%) and 20 as S2 subtype (56%). For the S1 subtype, the percentage of patients who reached PR, SD and PD was 68.75% (11/16), 18.75% (3/16) and 12.5% (2/16), respectively. For the S2 subtype, the percentage of patients who reached PR, SD and PD was 25.0% (5/20), 55% (11/20) and 20% (4/20), respectively. Overall, the accuracy of the 15-gene expression signature for the prediction of immunotherapy efficacy was 72.2% (68.8% sensitivity and 75% specificity). As shown in Figure 4C, the proportion of S1 subtype patients had a response rate that was nearly more than triple the proportion of S2 subtype patients (68.75% vs. 25%, Chi-Square *p* = 0.028).

We then compared the predictive values of a molecular subtype with other immunotherapy predictive biomarkers. The ImmuCellAI algorithm was applied to summarize the abundance of 24 immune cell types into an infiltration score and predict the response of immunotherapy for each sample. The ImmuCellAI predicted 27 patients as responders and 9 patients as non-responders (Supplementary Table S5). When compared with the clinical evaluation outcomes, the overall accuracy of ImmuCellAI predictions was 47.2%, with a sensitivity of 75% for predicting responsive patients and a specificity of 25% for predicting non-responsive patients. The TIDE algorithm applies a combination of transcriptomic signatures to estimate T cell dysfunction and T cell exclusion scores for predicting immunotherapy efficacy. TIDE predicted 30 patients as responders and 6 patients as non-responders (Supplementary Table S5). The overall accuracy of TIDE predictions was 50.0%, with a sensitivity of 87.5% for predicting responsive patients and a specificity of 20% for predicting non-responsive patients. Unsurprisingly, the waterfall plots showed that no significant difference in terms of ImmuCellAI-score and TIDE-score was observed between responder and non-responder groups (Figure 4D). Only the molecular subtype tended to have greater power for immunotherapy efficacy prediction, as revealed by the higher prediction score of the 15-gene expression signature in a non-responder group compared to a responder group (*t*-test *p*-value = 0.036, Figure 4D).

### 3.5. Drug Prediction for S2 Subtype ESCC Patients

**Figure 6:**
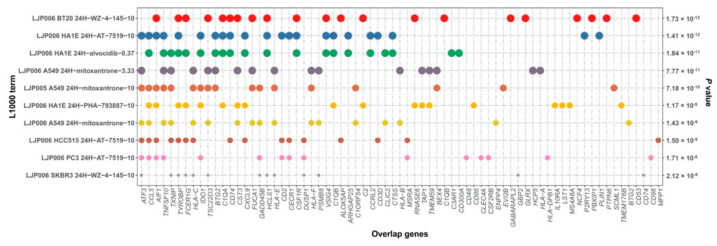
Bubble plot showing the distribution of the genes for each LINCS L1000 Chem Pert category. The color of the bubbles represents the L1000 term, and the size of the bubbles indicates the *p*-value of the terms.

Given the higher risk of tumor progression and lower response rate to immunotherapy, we sought to find novel treatment options for patients in the S2 subtypes. For the purpose of drug prediction, the list of 289 genes upregulated in the S2 subtype was retrieved, used as a query signature, and then mapped to the LINCS L1000 Connectivity Map resource. The LINCS L1000 project has collected gene expression profiles for thousands of perturbagens at a variety of time points, doses and cell lines. Comprehensive gene-drug interactions were profiled and curated in the “LINCS L1000 chem pert category”. The enrichment analysis of the “LINCS L1000 chem pert” category was performed by the Enrichr bioinformatics tool. Among the 289 genes, we found numerous drug compounds whose administration significantly changed the expression level of the selected genes. The top 10 significant perturbagens for LINCS L1000 Chem Pert up were provided in Figure 6 and Supplementary Table S6. Interestingly, mitoxantrone exhibited the fourth, fifth and seventh smallest *p*-values among the top 10 most significant perturbagens, suggesting this small molecular drug might be used and have potential clinical value for treating the S2 subtype of ESCC patients in addition to conventional therapies.

## 4. Discussion

ESCC is one of the deadliest cancers with high malignancy and poor outcomes. Although the combination of radiotherapy, surgery and chemotherapy is utilized in ESCC treatment, ESCC patients experience poor prognoses as before. In clinical practice, immunotherapy is being utilized to treat multiple cancer types, including ESCC, but only a small percentage of ESCC patients respond to immunotherapy [9,10,11,12]. It is necessary to have a better understanding of the intratumoral heterogeneity in cancer progression and the tumor-immune microenvironment to enhance immunotherapy response and outcomes. Novel biomarkers, especially immune-related gene signatures, are emerging to determine the molecular subtypes of cancer. While molecular subtyping has successfully guided clinical treatment for various cancers, related analysis for ESCC is rare. For instance, cell of origin subtyping for diffuse large B cell lymphoma and the 21-gene recurrence score for breast cancer are widely used in prognostic assessment and treatment guidance [33,34]. Therefore, identifying a prognostic and predictive immune-related signature is needed and is significant for ESCC. Such a signature might aid clinicians in determining the immune status of ESCC patients, serve as a prognostic factor for patient survival and allow immunotherapeutic efficacy stratification.

In the present study, by performing integrative analysis of gene expression and DNA methylation data, we identified two clinically and immunologically distinct subgroups of ESCC tumors. In the TCGA cohort, a significant prognostic impact was observed for the molecular subtypes (*p* < 0.005, Figure 2). The S1 subtype had a superior prognosis for OS by comparing it with the S2 subtype. Multivariate Cox regression analysis further confirmed the survival difference was independent of age, gender, smoke, alcohol, histopathological factors and clinical stages. A list of 305 genes differentially expressed between two subtypes were significantly associated with immune activities and immune regulations, suggesting the specified molecular subtype is not only associated with malignant cells but also impacted by the TME. Tumor-infiltrating immune cells in the TME are crucial for enhancing antitumor and immunotherapeutic effects. We applied the CIBERSORT ABS algorithm to assess the relationship between the molecular subtypes and abundance of 22 distinct immune cells in TME. In the results, the S2 subtype exhibited increased predicted proportions of Tregs, T cell CD4+ memory resting, T cell follicular helper, macrophages and activated mast cells compared with the S1 subtype (Wilcoxon *p* < 0.01). We also found that T cell exhaustion showed distinct patterns between two subtypes, characterized by significantly higher expression of immune inhibitory receptors (*CTLA4*, *LAG3*, *PDCD1*, *HAVCR2*, *TIGIT* and *TNFRSF9*) in the S2 subtype. Recently, Zheng et al. conducted single-cell gene expression profiling coupled with T cell receptor sequencing analyses to characterize the immune cells in seven pairs of ESCC tumors and matched adjacent tissues [35]. Their data showed that a continual progression of CD8 T cells from pre-exhausted to exhausted T cells. While exhausted CD4, CD8 T and NK cells constitute the bulk of the proliferative cell components in the TME, macrophages-Tregs crosstalk contributes to potential immunosuppression in the TME. Of interest, their findings are highly consistent with our data. It is rational to speculate that the seven ESCC tumors they analyzed likely belong to our S2 subtype.

These findings were then verified with the clinical investigation. We applied the molecular subtyping to an independent cohort of Chinese ESCC patients treated with ICIs therapies. Among 36 patients, 16 patients were assigned the S1 subtype (44%) and 20 the S2 subtype (56%). Significantly, the molecular subtypes were associated with patients’ responses to immunotherapies. We observed a much higher response rate to immunotherapies in Subtype S1 (11 of 16; 68.75%) than in Subtype S2 (5 of 20; 25%) patients (*p* = 0.028), suggesting that molecular subtypes exhibited distinct responsive patterns to ICIs therapies. This makes the defined molecular subtype a valuable predictive marker for immunotherapy administration. We further evaluated two popular algorithms, ImmuCellAI and TIDE, to predict patients’ responses to ICIs treatment. When compared with the clinical evaluation outcomes, the ImmuCellAI algorithm achieved an overall accuracy of 47.2% (75% sensitivity and 25% specificity) and the TIDE algorithm achieved an overall accuracy of 50.0% (87.5% sensitivity and 20% specificity) for predicting the responsive patients. Although sensitivity was satisfied, this was at the expense of specificity, suggesting these predictive markers failed to eliminate a significant proportion of patients who ultimately would not benefit from the ICIs therapies. Further validation studies of the utility of the defined molecular subtype in predicting immunotherapeutic efficacy are essential to confirm our results, which are based on modest-sized cohorts. Ideally, these future studies should be performed in a prospective manner.

We further seek optimal therapeutic strategies for S2 subtype patients based on the significantly up-regulated genes in the S2 subtype. Of interest, the candidate drug with the highest enrichment scores from the mapped compounds is mitoxantrone. Mitoxantrone (1,4-dihydroxy-5,8-bis[[2-[(2-hydroxyethyl)amino]ethyl]amino]-9,10-anthracenedione) is a synthesized antineoplastic drug. Its structure is similar to classical anthracyclines, which have fewer cardiotoxic effects than naturally occurring anthracyclines [36]. As an admitted anticancer drug, mitoxantrone has been used as a potent chemotherapeutic component against several cancer types. In multiple comparative trials of advanced breast cancer, mitoxantrone consistently showed good efficacy and comparable activity relative to doxorubicin, whereas it displayed significantly fewer toxicities [37,38]. Moreover, mitoxantrone has also displayed particular promise in the treatment of acute nonlymphocytic leukemia, acute lymphoblastic leukemia and acute myeloid leukemia when used alone or in conjunction with cytarabine [39,40,41,42]. Recently, mitoxantrone has been used to treat patients with advanced prostate cancer. In about 30% of hormone-refractory prostate cancer patients, mitoxantrone plus the corticosteroid prednisone shows a palliative benefit, which could reduce pain and analgesic usage [43,44]. To the best of our knowledge, very little data has been reported regarding the efficacy of mitoxantrone in esophageal cancer. Hoffmanns et al. performed a preliminary clinical study, which demonstrated the efficacy of mitoxantrone in five inoperable, recurrent esophageal carcinoma patients [45]. In each patient, they observed transitory subjective and objective responses. Meanwhile, the drug was well tolerated. No local or systemic side effects were investigated. Further in vitro and in vivo studies evaluating the effects of mitoxantrone in ESCC subtype patients remain a very interesting topic for future investigation.

The application of molecular biomarkers in ESCC has been reported in succession. For instance, Wang et al. reported a six-gene signature, and Mao et al. established a seven-lncRNA signature for survival prediction in ESCC [46,47]. A DNA methylation-related five-gene signature in ESCC was also identified [48]. Zhang et al. discovered immune-related subtypes to predict the survival and inflammatory landscapes of ESCC [49]. Recently, Gao et al. implemented Single-SampleGeneSet Enrichment Analysis to establish two ESCC subtypes (Immunity-High and Immunity-Low) and suggested the Immunity-High subtype may respond to immunotherapy because of higher expression of immune checkpoints, such as PD1, PD-L1, CTLA4 and CD80 [50]. Our work differs from those of previous studies in several significant aspects. First, we performed SNF-CC analysis to integrate the gene expression and DNA methylation profiles of ESCC. Cavalli et al. demonstrated that the information on gene expression and DNA methylation profiles is complementary [16]. Both datasets contribute significantly and differently in elucidating the true intertumoral heterogeneity. Therefore, the SNF-CC integrative analysis allows a unified view of the underlying structures that is valuable in discovering the molecular subtypes of ESCCs. Recently, Liu et al. performed a large-scale mass spectrometry-based proteomic and phosphoproteomic study of esophageal cancer and defined two clinically relevant molecular subtypes [51]. Therefore, future studies further integrating emerging technologies such as proteomics and histone modifications together with gene expression and DNA methylation profiling may therefore enable an even more accurate depiction of the ESCC molecular landscape.

Second, ESCC molecular subtypes will be able to be clinically translated with the development of subtype-specific diagnostic biomarkers. We employed a machine learning-based approach to identify and construct a 15-gene diagnostic model that could accurately classify ESCC subtypes. By doing so, we leveraged the prognostic and predictive power of molecular subtypes of ESCC into a concise gene expression signature, which may offer clinically important data for prognosis assessment and treatment planning. The panel included genes previously described to be involved in cytokine signaling in the immune system, B cell receptor signaling pathway, MAPK signaling pathway (*MAPK9*), apoptosis, cell cycle (*M1AP*, *CCDC69*), cell migration (*TBXT*), DNA damage response (*UIMC1*), regulation of gene transcription (*LIN28B*, *TSC22D1*, *TSN*), direct p53 effectors (*SCN3B*), oxidation-reduction process (*PHYHD1*) and other processes (*GPR137B*, *TMEM185B*, *SNHG28*, *FNDC9*, *LINC00847*), although some of these genes are involved in multiple additional pathways. In future work, we aim to translate the 15-gene expression signature into a convenient and reliable molecular assay that will allow the application to routinely available FFPE biopsy samples obtained at the time of tumor diagnosis.

Third, the ultimate aim of ESCC molecular subtype recognition is to develop subtype-specific treatment approaches and translate them into disease management. We believe that the defined molecular subtypes could have clinical implications for the development of combination treatment plans and guide the optimal selection of patients for immunotherapy. For patients with a favorable prognosis and an enhanced local immune phenotype (S1 subtype), immunotherapies may be utilized to boost the preexisting antitumor immunity of these patients and further improve their outcomes. However, for patients belonging to the S2 subtype, ICIs therapy alone may be insufficient due to the high density of exhausted T cells and/or the presence of immune-suppressive mechanisms. For the S2 subtype patients, the combination of immunotherapy with radiotherapy and chemotherapy, as well as other emerging treatment options, may be used to amplify or boost the inhibited immune response.

## 5. Conclusions

In summary, the present study offers a new perspective on the molecular subtyping of ESCC and the biological underpinnings of novel therapeutic interventions in a specific subgroup of ESCC that could potentially be translated into clinical settings both diagnostically and therapeutically to benefit ESCC patients significantly.

## Figures and Tables

**Table 1:** Clinical characteristics of TCGA-ESCC patients.

Characteristics	Total Cohort	S1 (%)	S2 (%)	χ^2^	*p*-Value
Number of patients	94	40	54		
Status					
Alive	75	36 (90.0)	39 (72.2)	3.468	0.062
Dead	19	4 (10.0)	15 (27.8)		
Gender					
Male	81	33 (82.5)	48 (88.9)	0.342	0.559
Female	13	7 (17.5)	6 (11.1)		
Age at diagnosis					
Mean	58	58	59		
Range	36–90	36–84	36–90		
Race					
Asian	45	21 (52.5)	24 (44.4)	3.365	0.339
White	41	16 (40.0)	25 (46.3)		
Black (black or African American)	5	3 (7.5)	2 (3.7)		
NA	3	0 (0.0)	3 (5.6)		
Tumor central location					
Mid	44	18 (45.0)	26 (48.1)	1.543	0.672
Distal	43	18 (45.0)	25 (46.3)		
Proximal	6	3 (7.5)	3 (5.6)		
Not specified	1	1 (2.5)	0 (0.0)		
Stage					
I	6	3 (7.5)	3 (5.6)	5.045	0.283
II	55	27 (67.5)	28 (51.9)		
III	27	9 (22.5)	18 (33.3)		
IV	4	0 (0.0)	4 (7.4)		
NA	2	1 (2.5)	1 (1.8)		
Grade					
Grade 1	16	11 (27.5)	5 (9.3)	5.806	0.122
Grade 2	48	17 (42.5)	31 (57.4)		
Grade 3	21	9 (22.5)	12 (22.2)		
Grade X	9	3 (7.5)	6 (11.1)		
Alcohol					
Yes	68	31 (77.5)	37 (68.5)	1.136	0.567
Never	24	8 (20.0)	16 (29.6)		
NA	2	1 (2.5)	1 (1.9)		
Smoking					
Never	32	15 (37.5)	17 (31.5)	0.896	0.826
Current	28	10 (25.0)	18 (33.3)		
Reformed ≤ 15 years	21	8 (20.0)	13 (24.1)		
Reformed > 15 years	9	4 (10.0)	5 (9.3)		
NA	4	3 (7.5)	1 (1.8)		
Radiation treatment					
Yes	30	14 (35.0)	16 (29.6)	4.140	0.126
No	40	20 (50.0)	20 (37.1)		
NA	24	6 (15.0)	18 (33.3)		
Pharmaceutical treatment					
Yes	8	5 (12.5)	3 (5.5)	1.676	0.433
No	69	29 (72.5)	40 (74.1)		
NA	17	6 (15.0)	11 (20.4)		

**Table 2:** Cox proportional-hazard regression analysis for different characteristics in ESCC patients.

Characteristics	Univariate Analysis			Multivariate Analysis		
	Hazard Ratio	95% CI	*p*-Value	Hazard Ratio	95% CI	*p*-Value
Gender						
Male	Reference					
Female	0.14	0.02–1.00	5.01 × 10^−2^	0.35	0.03–4.00	4.02 × 10^−1^
Age at diagnosis	1.03	0.99–1.08	1.26 × 10^−1^	1.03	0.97–1.11	3.07 × 10^−1^
Race						
Asian	Reference					
White	1.53	0.65–3.63	3.31 × 10^−1^	1.37	0.45–4.16	5.80 × 10^−1^
Black (black or African American)	3.05	0.81–11.44	9.89 × 10^−2^	0.18	0.01–3.20	2.40 × 10^−1^
Tumor central location						
Mid	Reference					
Distal	0.81	0.38–1.74	5.92 × 10^−1^	1.51	0.53–4.28	4.36 × 10^−1^
Proximal	0	0–Inf	9.98 × 10^−1^	0	0-Inf	9.98 × 10^−1^
Stage						
I–II	Reference					
III–IV	2.59	1.23–5.46	1.26 × 10^−2^	1.25	0.46–3.39	6.59 × 10^−1^
Histologic grade						
Grade 1	Reference					
Grade 2	1.84	0.62–5.46	2.71 × 10^−1^	0.61	0.15–2.47	4.89 × 10^−1^
Grade 3	0.93	0.23–3.71	9.13 × 10^−1^	0.24	0.05–1.20	8.24 × 10^−2^
Alcohol consumption						
Never	Reference					
Yes	2.02	0.7–5.85	1.93 × 10^−1^	1.57	0.39–6.27	5.2 × 10^−1^
Tobacco smoking history						
Never	Reference					
Yes	1.51	0.64–3.55	3.46 × 10^−1^	0.74	0.21–2.62	6.4 × 10^−1^
Molecular types						
Subtype 1	Reference					
Subtype 2	13.53	3.85–47.57	4.91 × 10^−5^	51.60	3.99–667.48	2.5 × 10^−3^

**Table 3:** Clinical characteristics of ESCC patients in the validation set.

Characteristics	Total	S1 (%)	S2 (%)	χ^2^	*p*-Value
Number of patients	36	16	20		
Gender					
Male	31	14 (87.5)	17 (85.0)	6.390	1.000
Female	5	2 (12.5)	3 (15.0)		
Age at diagnosis					
Median	64.5	63.5	65		
Range	47–79	52–79	47–79		
Stage					
II	8	4 (25.0)	4 (20.0)	4.950	0.084
III	12	8 (50.0)	4 (20.0)		
IV	16	4 (25.0)	12 (60.0)		
Differentiation ^1^					
Well	5	3 (30.0)	2 (16.7)	0.054	0.816
Moderately/poorly	17	7 (70.0)	10 (83.3)		
Alcohol ^2^					
Yes	16	9 (56.2)	7 (38.9)	0.446	0.504
Never	18	7 (43.8)	11 (61.1)		
Smoking ^3^					
Never	18	6 (37.5)	12 (66.7)	4.183	0.124
Current	14	8 (50.0)	6 (33.3)		
Reformed ≤ 15 years	0	0 (0.0)	0 (0.0)		
Reformed > 15 years	2	2(12.5)	0(0.0)		

^1^ The differentiation of 14 patients are not defined. ^2^ The alcohol status of 2 patients are not available. ^3^ The smoking status of 2 patients are not available.

## Data Availability

The datasets generated and/or analyzed during the current study are available in the National Genomics Data Center repository (https://ngdc.cncb.ac.cn/, accessed on 13 May 2022). Accession: HRA002386.

## Supplementary Materials

The following supporting information can be downloaded at: https://www.mdpi.com/article/10.3390/cancers14204970/s1. Table S1: Upregulated and downregulated genes in the S2 subtype compared to S1 subtype. Table S2: GO category analysis of 305 genes. Table S3: KEGG pathways analysis of 305 genes. Table S4: List of selected 15 genes and related subtype. Table S5: Immunotherapy efficacy prediction of TIDE and ImmuCellAI. Table S6: Top 10 significant *p*-values and q-values for LINCS L1000 Chem Pert up.
